# Temperature-Dependent Synergistic Effect of Multi-Walled Carbon Nanotubes and Graphene Nanoplatelets on the Tensile Quasi-Static and Fatigue Properties of Epoxy Nanocomposites

**DOI:** 10.3390/polym13010084

**Published:** 2020-12-28

**Authors:** Yi-Ming Jen, Hao-Huai Chang, Chien-Min Lu, Shin-Yu Liang

**Affiliations:** Department of Mechanical and Mechatronic Engineering, National Taiwan Ocean University No. 2, Pei-Ning Rd., Keelung 20224, Taiwan; how0524@gmail.com (H.-H.C.); z0937839664@gmail.com (C.-M.L.); l0910784791@gmail.com (S.-Y.L.)

**Keywords:** temperature effect, synergistic effect, carbon nanotube, graphene nanoplatelet, epoxy nanocomposites, quasi-static property, fatigue property

## Abstract

Even though the characteristics of polymer materials are sensitive to temperature, the mechanical properties of polymer nanocomposites have rarely been studied before, especially for the fatigue behavior of hybrid polymer nanocomposites. Hence, the tensile quasi-static and fatigue tests for the epoxy nanocomposites reinforced with multi-walled carbon nanotubes (CNTs) and graphene nanoplatelets (GNPs) were performed at different temperatures in the study to investigate the temperature-dependent synergistic effect of hybrid nano-fillers on the studied properties. The temperature and the filler ratio were the main variables considered in the experimental program. A synergistic index was employed to quantify and evaluate the synergistic effect of hybrid fillers on the studied properties. Experimental results show that both the monotonic and fatigue strength decrease with increasing temperature significantly. The nanocomposites with a MWCNT (multi-walled CNT): GNP ratio of 9:1 display higher monotonic modulus/strength and fatigue strength than those with other filler ratios. The tensile strengths of the nanocomposite specimens with a MWCNT:GNP ratio of 9:1 are 10.0, 5.5, 12.9, 23.4, and 58.9% higher than those of neat epoxy at −28, 2, 22, 52, and 82 °C, respectively. The endurance limits of the nanocomposites with this specific filler ratio are increased by 7.7, 26.7, 5.6, 30.6, and 42.4% from those of pristine epoxy under the identical temperature conditions, respectively. Furthermore, the synergistic effect for this optimal nanocomposite increases with temperature. The CNTs bridge the adjacent GNPs to constitute the 3-D network of nano-filler and prevent the agglomeration of GNPs, further improve the studied strength. Observing the fracture surfaces reveals that crack deflect effect and the bridging effect of nano-fillers are the main reinforcement mechanisms to improve the studied properties. The pullout of nano-fillers from polymer matrix at high temperatures reduces the monotonic and fatigue strengths. However, high temperature is beneficial to the synergistic effect of hybrid fillers because the nano-fillers dispersed in the softened matrix are easy to align toward the directions favorable to load transfer.

## 1. Introduction

Adding an individual type of carbon nano-particle in the polymer matrix has been confirmed in numerous past studies to improve the mechanical, thermal, and electrical properties of the polymer materials, and some review studies are available for references [1,2,3,4]. In general, adding an appropriate amount of carbon nano-fillers in polymer materials can display significant enhancement on various properties of polymers, nevertheless, the agglomeration caused by the addition of excessive nano-reinforcements is detrimental to the properties needed [5,6,7]. The characteristic morphology of utilized nano-particles [8], size of particles [9,10], and interfacial strength between the particle and matrix [11] are found to be the important factors to influence the dispersion of the nano-fillers in the polymer matrix markedly.

The carbon nano-reinforcements can be classified according to their special structure dimensionality. The particle-like nano-fillers, such as fullerene, carbon black, etc., are classified as zero-dimensional (0-D) reinforcements. The carbon nanotube (CNT) family, such as single-walled CNTs (SWCNTs), double-walled CNTs (DWCNTs), and multi-walled CNTs (MWCNTs), are named as 1-D reinforcements. The 2-D reinforcements are the ones with flaky or layered structures, such as the graphene-based nano-fillers. Physically, the nano-fillers with higher dimensionality provide larger contact surface with the polymer matrix [12,13,14,15], however the π-π interaction and van der Waals force in the nano-structure of the employed reinforcements are likely to stimulate the agglomeration of the nano-fillers, further reducing the properties significantly [16,17]. Accordingly, many chemical and physical techniques have been developed to improve the dispersion of the nano-fillers in the polymer matrix and enhance the interfacial crosslink between the filler and the matrix [18,19,20,21,22]. Employment of a second phase of nano-filler in the polymer matrix is another way to solve the problem of filler agglomeration [23,24,25]. By taking advantage of different morphological characteristics of two types of nano-fillers, the problem of agglomeration can be solved, and the studied properties can be improved. Among the nanocomposites reinforced with all possible combinations of carbon nano-fillers, the hybrid nanocomposites with CNTs and graphene-family nano-sheets have received much attention because the CNTs between the flaky graphene-based reinforcements can prevent the flaky nano-particles from agglomeration [26,27,28]. Furthermore, the flexible tube-like CNTs and the planar graphene-based reinforcements constitute an effective 3-D network within the polymer matrix, providing an excellent bridging effect on the load, thermal, and electrical transfer.

Recently, some studies regarding the synergistic effect of CNTs and graphene-based nano-sheets on the characteristics of polymer have been performed. Among the investigated properties, the mechanical property has attracted much interest because the nanocomposite technology developed in laboratories has been widely applied in structural applications in the past decade. For examples, the carbon nano-particles have been embedded in the polymer matrix directly to improve the bulk or interlaminar strengths of the traditional fiber-reinforced laminates [29]. Inserting nanocomposite interlayers between the prepregs is another technique to resist the delamination damage of the fiber reinforced structures. The carbon nano-particles have also been added in the polymer adhesives in the automobile and aircraft industries to improve the adhesion strengths [30]. Surveying the past studies relevant to the synergistic effect of CNTs and graphene-based nano-sheets on the mechanical properties of polymer nanocomposites demonstrates that the analyzed mechanical properties in these previous research works are focused on the quasi-static tensile properties [24,25,31,32,33,34,35,36,37,38,39,40,41,42,43,44,45], quasi-static flexural properties [25,44,46,47], viscoelastic properties [48,49], and fracture toughness [25,32,40,46,47]. For the aforementioned trinary nanocomposite studies, the filler ratio between the hybrid reinforcements was the most critical variable to obtain the nanocomposites with optimal mechanical properties. Despite divergent results of the optimal filler ratios for the studied mechanical properties being reported, most related studies show that the nanocomposites with extreme filler ratios (rich CNTs or rich graphene nanoplatelets (GNPs)) display a higher synergistic effect of hybrid nano-fillers than those with other filler ratios. The hybrid nanocomposites with rich CNTs and few graphene-based nano-sheets were reported to have optimal mechanical properties among the ones with various filler ratios [24,26,31,44,46,47,49]. Oppositely, experimental results of some studies show that the nanocomposites with plentiful graphene-based particles and tiny amounts of CNTs have distinctive mechanical properties among the ones with different filler ratios [24,43]. Furthermore, it is noted that the fatigue property of the polymer nanocomposites reinforced with CNTs and graphene-based fillers was rarely investigated till 2020. Jen et al. [50] studied the synergistic effect of MWCNTs and graphene nanoplatelets (GNPs) on the fatigue life and fatigue crack propagation rate of the epoxy nanocomposites with various filler ratios. The nanocomposites with a MWCNT: GNP of 1:9 was found to have higher fatigue strength and lower fatigue crack growth rate than those with other filler ratios.

Another common feature in surveying the past experimental studies regarding the mechanical properties of the polymer nanocomposites with hybrid nano-fillers is that the majority of utilized tests for investigated properties have been performed at room temperature. In order to expand the utilization of the innovative nanocomposites, understanding the temperature-dependent mechanical properties of the nanocomposites is important in the fields of design and application because the performance of polymer materials is sensitive to ambient temperature. Some efforts have been made to investigate the temperature effect on the mechanical properties of polymer nanocomposites reinforced with individual types of carbon nano-filler, such as clay [51,52,53], CNTs [54,55,56,57,58,59], and graphene oxide [60]. The considered temperature-dependent mechanical characteristics in these studies were focused on the monotonic [52,53,54,55,58,59,60], viscoelastic [55,57,58,59,60], or impact properties [51,56]. In general, the studied mechanical strengths of the nanocomposites decrease with increasing temperature. Since engineering materials are constantly subjected to cyclic loading in adverse environments, the temperature-dependent knowledge of the fatigue behavior for the polymer nanocomposites is extremely important in application. The available references regarding the fatigue strength of the nanocomposites with hybrid nano-fillers are rare. Jen et al. [61] studied the influence of temperature on the fatigue behavior of the epoxy nanocomposites reinforced with CNTs in 2014. Both the static and fatigue strengths of the studied nanocomposites were found to decrease remarkably with increasing temperature. At each controlled temperature, the optimal loading of CNTs employed in the specimen preparation to obtain the highest fatigue strength is 0.5 wt.%.

Until now, the temperature effect on the synergistic effect of hybrid nano-fillers on the fatigue strength of polymer nanocomposites is not fully understood. Therefore, the aim of this study is to analyze experimentally the quasi-static and fatigue properties of epoxy nanocomposites reinforced with MWCNTs and GNPs at various temperatures and explore the temperature-dependent synergistic effect of the two employed nano-fillers on the studied mechanical properties. The filler ratios between hybrid fillers employed in the specimen preparation and the controlled temperatures were two main variables considered in the experimental program. Additionally, the fracture surfaces of the studied nanocomposite specimens were observed microscopically to verify the reinforcement mechanisms of the studied mechanical properties at different temperatures.

## 2. Materials and Methods

### 2.1. Materials and Specimen Preparation

The MWCNTs used in this study were supplied by Applied Nanotechnologies, Inc., Austin, TX, USA. The diameter and the length of the employed MWCNT are ranged from 20 to 40 nm and from 10 to 20 μm, respectively. The utilized GNPs with the designation of KNG-150 were purchased from Kano Co., Xiamen, China. The diameter of the GNP is approximately 1–20 μm, and the thickness is about 5–15 nm. Both the purities of employed CNTs and GNPs are larger than 99.5%. The solvent-type epoxy, designated EPO-622 and fabricated by Epotech Composite Co., Taiwan, was utilized to prepare the matrix material. The as-received epoxy solvent contains epoxy resin, dicyanamide curing agent, and methyl ethyl ketone (MEK) solvent. The weight ratio between the epoxy resin and curing agent is 18:2.

**Figure 1:**
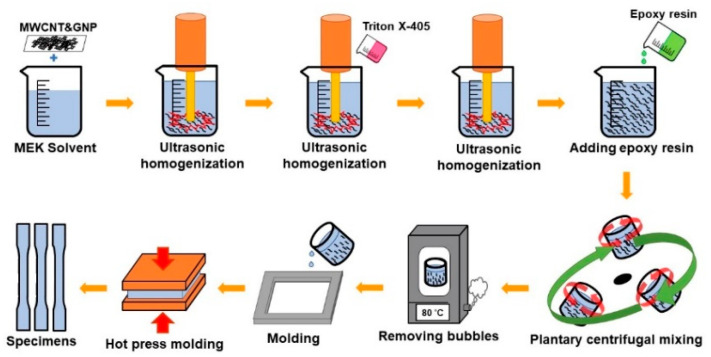
Procedure of preparation of nanocomposite specimens with carbon nanotube (CNT) and graphene nanoplatelet (GNP) hybrids.

**Figure 2:**
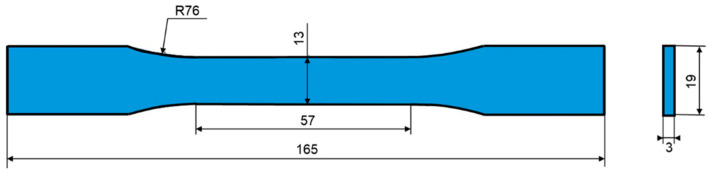
Shape and the dimensions of the specimen (unit: mm).

The procedure of preparing the specimens is illustrated in Figure 1. The MWCNTs and GNPs were heated at 120 °C for 1 h first to remove the moisture. The dried nano-fillers with required weight ratio were added in the MEK solvent. The suspension was then agitated for 20 min using an ultrasonic homogenizer. Next, the surfactant Triton X-405 (Sigma-Aldrich, Inc., St. Louis, MI, USA) was added in the mixture and the solution was agitated continuously for 10 min using an ultrasonic homogenizer to obtain the uniform dispersion of nano-fillers in MEK. Afterwards the suspension was mixed with the solvent-type epoxy using a planetary centrifugal mixer for 20 min. The mixture was then heated in a vacuum oven at 80 °C for 90 min to remove the MEK and bubbles from the blend. Then the mixture was poured into a mold and remained heated in a vacuum oven for 90 min. Next the blend was cured by hot-pressing at 150 °C with applying the pressure from 0 to 1000 psi gradually to obtained the solidified nanocomposites. The solidified composite plate was cut into the specimens according to the required shape and dimensions specified by the ASTM (American Society for Testing and Materials) standard D638 [62]. The shape and dimensions of the specimen are illustrated in Figure 2.

The total content of the employed nano-fillers for all types of specimens was kept constant at 0.25 wt.%. In a preliminary study, the tensile strengths of the CNT/epoxy and GNP/epoxy nanocomposites with the filler loading increased at an interval of 0.05 wt.% were tested, respectively. At low loadings of nano-fillers, the tensile strengths of both types of nanocomposites increase with the filler loading until the nanocomposites display the highest strengths. Because of the agglomeration of nano-fillers, the tensile strengths of the nanocomposites decrease when the employed filler contents exceed the optimal values. Experimental results show that both the optimal filler contents for CNT/epoxy and GNP/epoxy nanocomposites are 0.25 wt.%. Hence, 0.25 wt.% was selected as the controlled total content of two employed nano-fillers to analyze the mechanical properties of hybrid nanocomposites. Moreover, seven types of specimens with various MWCNT: GNP weight ratios, i.e., 0:0, 0:10, 1:9, 3:7, 5:5, 7:3, 9:1, and 10:0, were prepared to study the effect of filler ratios on the studied temperature-dependent mechanical properties. The specimens with the filler ratio of 0:0 represent the ones of neat epoxy. The specimens with the filler ratios of 0:10 and 10:0 mean the ones reinforced with only GNPs and CNTs, respectively. Moreover, the specimens with filler ratios of 1:9, 3:7, 5:5, 7:3, and 9:1 were prepared and tested to investigate the effect of filler ratios between two types of reinforcements on the studied mechanical properties.

### 2.2. Experimental Methods

**Figure 3:**
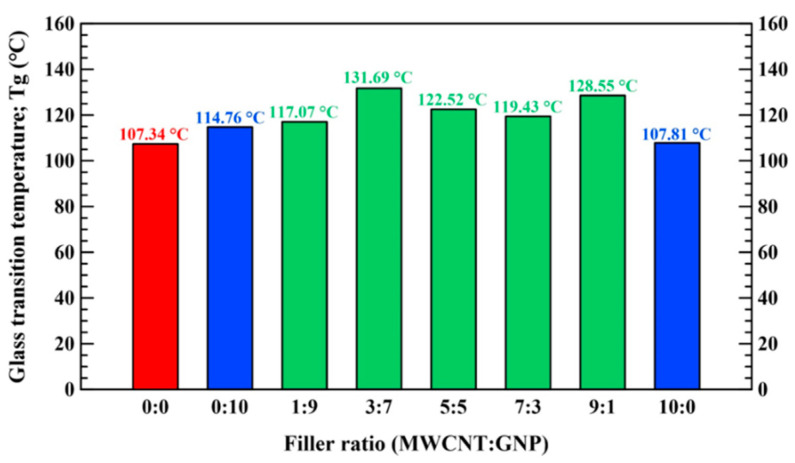
Experimental results of glass transition temperatures for the studied nanocomposite specimens with various filler ratios.

The tensile quasi-static and fatigue tests were performed according to ASTM standards D638 [62] and D7791 [63], respectively. All the tensile quasi-static and fatigue tests were conducted using an MTS 810 servo-hydraulic material system (MTS Systems Corporation; Eden Prairie, MN, USA) with a temperature-controlled chamber. The magnitude of error in controlling the temperature of the utilized chamber is approximately ±1 °C. The monotonic and fatigue tests for each type of nanocomposite specimens with different filler ratios were performed at five different temperatures, i.e., −28, 2, 22, 52, and 82 °C. The maximum controlled temperature was selected under the glass transition temperatures *T_g_* of all types of studied nanocomposites to avoid the softening of the specimens. Hence, the thermo-mechanical properties of all types of studied nanocomposite specimens with various filler ratios were analyzed using a dynamic mechanical analyzer (STA6000, PerkinElmer, UK) to obtain the glass transition temperatures of the nanocomposites. Figure 3 shows the variation of glass transition temperatures for all types of nanocomposite specimens with the filler ratios. It is evident that the glass transition temperature of neat epoxy is about 107.3 °C, and the glass transition temperatures of other types of specimens are all higher than 107.3 °C. Furthermore, all types of specimens began to lose the stiffness when the ambient temperature was higher than 90 °C. Hence, the temperature of 82 °C was selected as the maximum controlled temperature condition in the experimental program. The quasi-static tests were performed by controlling the stroke of crosshead at 0.02 mm/s. The engineering stress *σ* was obtained by dividing the applied load by the cross-sectional area at the gauge length of specimen. An extensometer with 20 mm gauge length (632.31F-24, MTS Systems Corporation; Eden Prairie, MN, USA) was applied to measure the tensile strain *ε*. For each type of nanocomposite specimens, three specimens were monotonically tested under the same temperature condition to obtain reliable experimental data. The fatigue tests were carried out under load control mode with the stress ratio *R* (defined as the minimum stress *σ*_min_ to the maximum stress *σ*_max_ in one cycle) of 0.1. The fatigue tests for each type of nanocomposite specimen with different filler ratios were conducted at five selected loading levels *r*, which was defined as the ratio of the maximum applied stress *σ*_max_ to the ultimate strength of the studied specimen *σ*_ult_ at the controlling temperature. The shape and frequency of the load waveforms are sinusoidal and 10 Hz, respectively. In order to study the influence of applied frequency, the temperature at the specimen surface was monitored using an infrared thermometer (8877AZ, AZ Instrument Corp., Taichung, Taiwan) during the fatigue test performed at room temperature. The temperature of a neat-epoxy specimen was measured when fatigue-tested at highest loading level (*r* = 70%) with frequency of 10 Hz. The type of specimen and the loading level were selected for the large plastic deformation caused by the specimen ductility and high load was expected. The temperature rise was found to increase gradually and reach the highest value of 4.8 °C when the specimen fractured. Another measurement was performed on a specimen of same type at 55% loading level. The temperature rise increased gradually and reached a stable value of 3.4 °C at about 10^3^ cycles, and further remained constant till 10^6^ cycles. This tiny increase in temperature within 5 °C has a slight influence on the studied fatigue properties. The fatigue life *N_f_* was defined as the number of cycles corresponding to the specimen separation. The fatigue test was interrupted when the number of cycles exceeds one million, and the specimen was recognized to have infinite life. After the monotonic and fatigue tests, a field emission scanning electron microscope (SEM) (JSM-6330F, JEOL Ltd., Tokyo, Japan) was used to observe the fracture surfaces of specimens to identify the temperature-dependent reinforcement mechanism of the applied hybrid fillers system.

## 3. Results and Discussion

### Temperature-Dependent Monotonic Property

**Figure 4:**
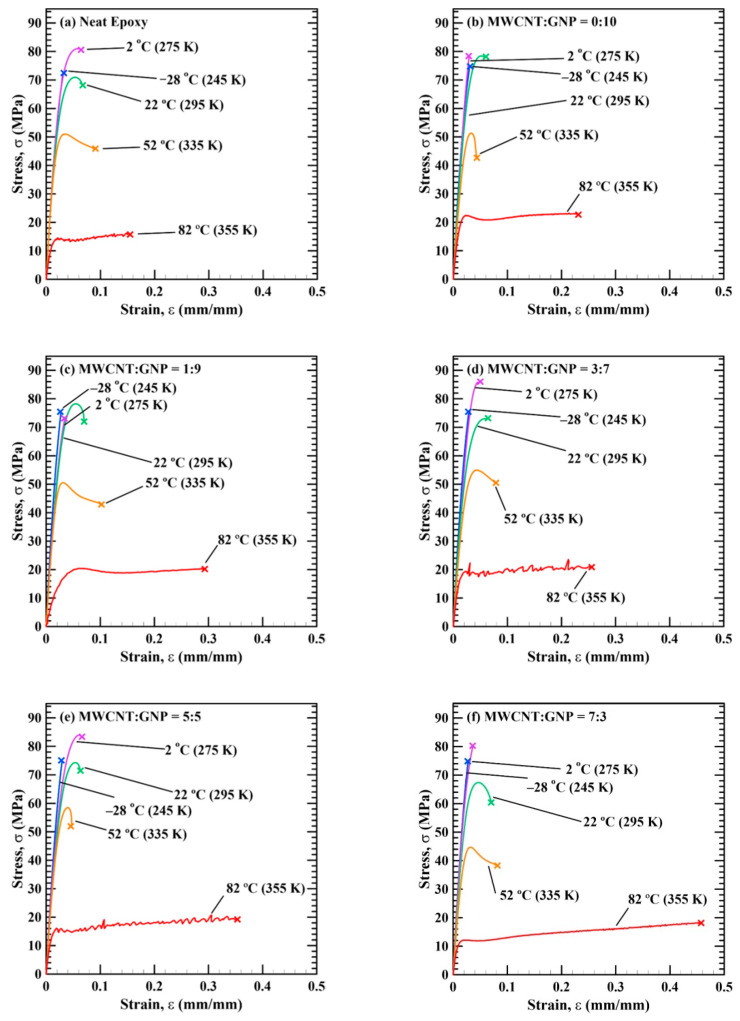

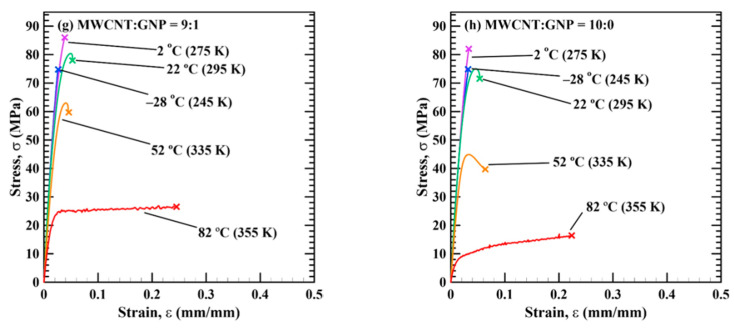
Stress–strain curves obtained in the quasi-statically tensile tests at various temperatures for the studied nanocomposites with the MWCNT:GNP ratios of (**a**) 0:0, (**b**) 0:10, (**c**) 1:9, (**d**) 3:7, (**e**) 5:5, (**f**) 7:3, (**g**) 9:1, and (**h**) 10:0.

Figure 4 illustrates the stress–strain curves of the studied nanocomposite specimens obtained in the quasi-statically tensile tests at various temperatures. It shows that the nanocomposite specimens display brittle characteristics when the ambient temperature is below the room temperature. These specimens express linear feature from the beginning of the tests, and fracture rapidly after slight nonlinear feature. Oppositely, the ductile characteristics of the nanocomposite specimens becomes more evident with increasing temperature when the environmental temperature is higher than the room temperature. The nonlinear section of the monotonic curves and the elongations of the specimens increase with the temperature significantly.

**Figure 5:**
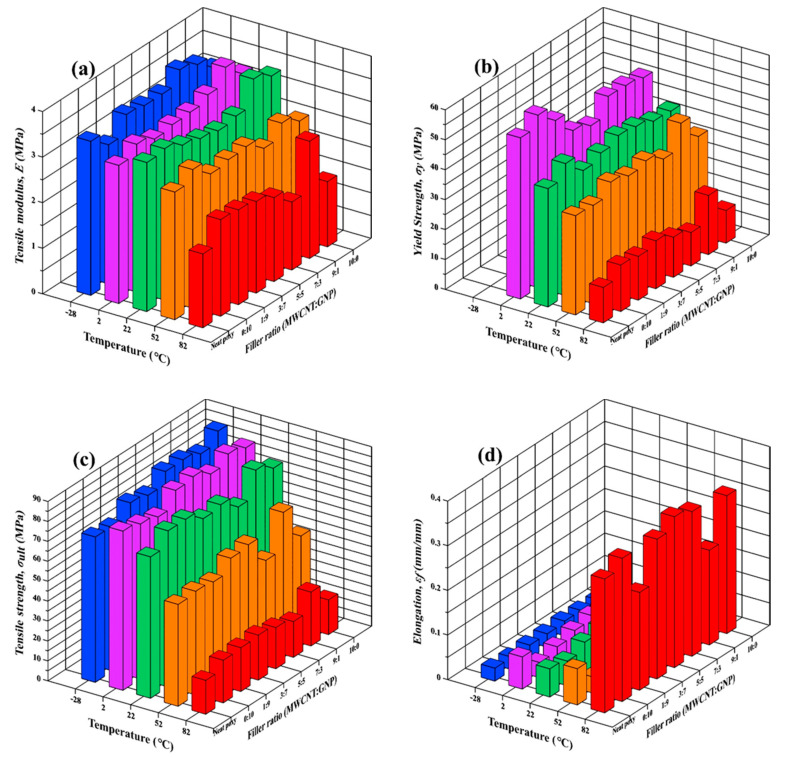
Variation of (**a**) tensile modulus, (**b**) yield strength, (**c**) ultimate strength, and (**d**) elongation of the studied nanocomposites with the temperature and the filler ratios.

Table 1 lists the monotonic properties obtained from the tensile tests performed at different temperatures. It is noted that there are no data of yield strength available for the specimens with evident brittleness because the yield strength here was obtained using the 0.2% offset criterion. Figure 5 shows the variation of the monotonic properties, i.e., tensile modulus *E*, yield strength *σ_y_*, tensile strength *σ_ult_*, and fracture elongation *ε_f_*, with the ambient temperatures and the filler ratios employed in preparation of nanocomposites. To avoid confusion, only average values of the studied mechanical properties are shown in this bar chart figure, and the presentation of standard deviations is omitted for clear visualization. The tensile modulus and tensile strength for all types of specimens almost remain constant or decrease slightly with increasing temperature when the temperature is lower than room temperature. However, the specimens lose stiffness and strength with increasing temperature significantly when the ambient temperature is higher than 22 °C. Furthermore, the yield strength of all types of nanocomposite specimens decreases gradually when the temperature rises from −2 to 82 °C. The elongation of the specimen slightly increases with the temperature until 52 °C. A remarkable increase in elongation was observed when the temperature rose from 52 to 82 °C.

Taking the employed number of filler types and filler ratios into consideration, the nanocomposites with only CNTs display higher tensile modulus and strength than those with only GNPs at low temperatures. The opposite trend is observed that at high temperatures, adding GNPs in epoxy matrix is more effective than adding GNPs in improving the monotonic modulus and strength. It implies that GNPs are more difficult to agglomerate at high temperatures than at low temperatures, and the large contact surface between the GNPs and matrix is beneficial to the interfacial bonding, further improves the studied monotonic strength. Furthermore, the trinary nanocomposites with some specific filler ratios present higher modulus and strength than those with individual types of nano-filler. It is noted that the nanocomposites with a MWCNT:GNP ratio of 9:1 demonstrate higher tensile strength than the ones with other filler ratios under most controlled temperature conditions. Moreover, when the temperature rises from 22 °C to 82 °C, the increase rate of the tensile strength (based on that of neat epoxy) for the nanocomposites with a MWCNT:GNP ratio of 9:1 rises from 2 to 59%. The nanocomposites with a MWCNT:GNP ratio of 9:1 also show a high increase rate of the yield strength at 52 and 82 °C when compared with those of neat epoxy at the same temperatures (24 and 67%). A tremendous increase rate (59%) was observed for the tensile modulus of the nanocomposites with a MWCNT:GNP ratio of 9:1. The results mentioned above reveal that the synergistic effect on the modulus and strength for the nanocomposites with the filler ratio of 9:1 intensifies obviously at high temperatures.

A synergistic index proposed by Jen et al. [50] was used to evaluate the synergistic effect of a hybrid filler system on the studied properties. The index is defined as the percentage increase of studied property compared with the predicted property. The predicted property of the hybrid filler nanocomposite is calculated from the properties of the nanocomposites with individual types of nano-filler based on the weight ratio of nano-fillers. The detailed concept of the synergistic index can be referred to in [50], and the synergistic index *η* for the nanocomposite with a MWCNT:GNP ratio of *x*:*y* (*x* + *y* = 10) at a specific temperature can be expressed as

(1) $$\eta \;\left(\%\right)=\frac{P_{hybrid}-\left(\frac{P_{CNT}x\;+\;P_{GNP}y}{10}\right)}{\frac{P_{CNT}x\;+\;P_{GNP}y}{10}}\times 100$$

where *P_hybrid_*, *P_CNT_*, and *P_GNP_* represents the magnitudes of the studied properties for the nanocomposites with hybrid fillers, MWCNTs only (MWCNT: GNP = 10:0), and GNPs only (MWCNT: GNP = 0:10) at the same controlled temperature, respectively.

**Figure 6:**
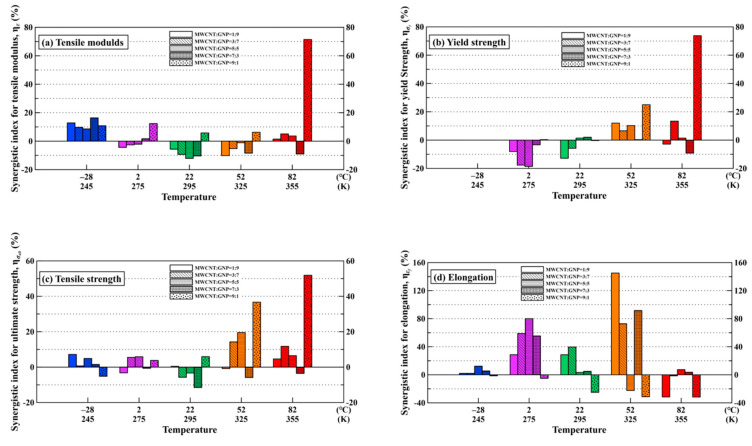
Variation of the synergistic indexes of CNTs and GNPs on the (**a**) tensile modulus, (**b**) yield strength, (**c**) tensile strength, and (**d**) elongation of the studied nanocomposites with the temperatures.

The synergistic indexes for the studied nanocomposite specimens at various temperatures are listed in Table 2. Figure 6 show the variation of synergistic indexes for the monotonic properties of the studied nanocomposites with the temperatures. It is obvious that the proposed index is a highly discriminating tool to quantify the synergistic effect of the hybrid fillers on the studied monotonic properties. The larger index signifies stronger synergistic effect. Moreover, the positive and negative indexes indicate beneficial and detrimental effects of employment of CNTs and GNPs on the studied property of epoxy, respectively. The nanocomposites with a MWCNT:GNP ratio of 9:1 show higher synergistic indexes on the modulus and strengths than the ones with other filler ratios, indicating that the nanocomposites with this specific filler ratio a demonstrate stronger synergistic effect on the studied properties. The high indexes obtained at high temperatures indicate that the synergistic effect increases with temperature. The tortuous CNTs between the layered GNPs provide a beneficial bridging effect on load transfer. The softening of polymer matrix at high temperatures facilitates the alignment of nano-fillers in the favorable direction of load transfer, further improves the stiffness and strength of the nanocomposites. Furthermore, comparing Figure 6c,d demonstrates a trade-off between the strength and elongation of the studied nanocomposites.

Figure 6 also shows that the epoxy nanocomposites with a filler ratio of 7:3 have much lower synergistic effect on the tensile modulus, yield strength, and tensile strength when compared with those with other filler ratios. For the nanocomposite specimens with the filler ratio of 9:1, the CNTs constitute bridging network between the GNPs to avoid the agglomeration of GNPs and improve the load transfer. However, the amount of GNPs for the nanocomposites with the filler ratio of 7:3 is higher than that with the filler ratio of 9:1, implying that the high concentration of GNPs is easy to cause agglomeration of GNPs due to the π-π interaction and van der Waals force within the layered structure of GNPs. Furthermore, excessive GNPs also obstruct the dispersion of CNTs, leading to the low stiffness and strength of the nanocomposites.

**Figure 7:**
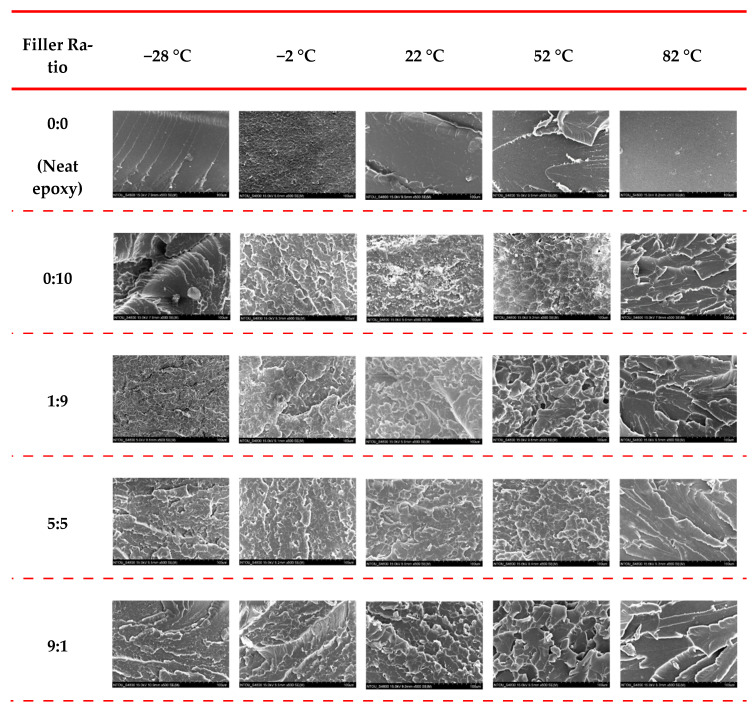

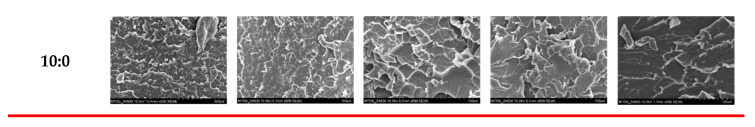
Low magnification SEM micrographs (magnification 500×) of the fracture surfaces of the studied nanocomposite specimens obtained at different temperatures.

The low magnification SEM micrographs (500×) of the monotonic fracture surfaces of the studied nanocomposite specimens obtained at different temperatures are shown in Figure 7. It is evident that the characteristics of fracture surfaces change from rough to smooth features as the temperature increases. Several previous works performed at room temperature have revealed that the cleavage surfaces are the important evidence for the enhancement mechanism on the mechanical strength of polymer nanocomposites reinforced by CNTs [64,65] and graphene-based particles [66,67]. When the crack front encounters the obstacle of nano-fillers, the crack plane must deflect the growth direction to bypass the cluster of nano-fillers. This mechanism requires more energy consumed and further improves the mechanical properties. Zhou et al. [64] demonstrated that the better the nano-fillers disperse, the greater number and the smaller size of the cleavage planes are observed. Furthermore, the reinforcement mechanism is more effective when the height difference between the cleavage planes increases [50,65]. As shown in Figure 7, for each type of nanocomposite, the size of cleavage planes becomes larger when the temperature increases, indicating that the fewer obstacles encountered, the less energy dissipated. At high temperatures, the nano-fillers are pulled out from the softened matrix with less difficulties, and accordingly, the fracture planes bypass the nano-filler obstacles more easily. The mechanism of cleavage surfaces and crack deflection effect fully explain that the monotonic strength of the studied nanocomposites decreases with increasing temperature.

Figure 7 also shows that at each specific temperature, the fracture surface of the specimen with a MWCNT:GNP ratio of 9:1 shows smaller cleavage planes when compared with those with other filler ratios. Furthermore, the height difference between the cleavage planes of the specimen with a filler ratio of 9:1 is larger than those with other filler ratios, meaning that the rougher fracture surfaces are observed. The mechanism also clarifies why the nanocomposites with a MWCNT:GNP ratio of 9:1 has a higher monotonic strength than those with other filler ratios.

**Figure 8:**
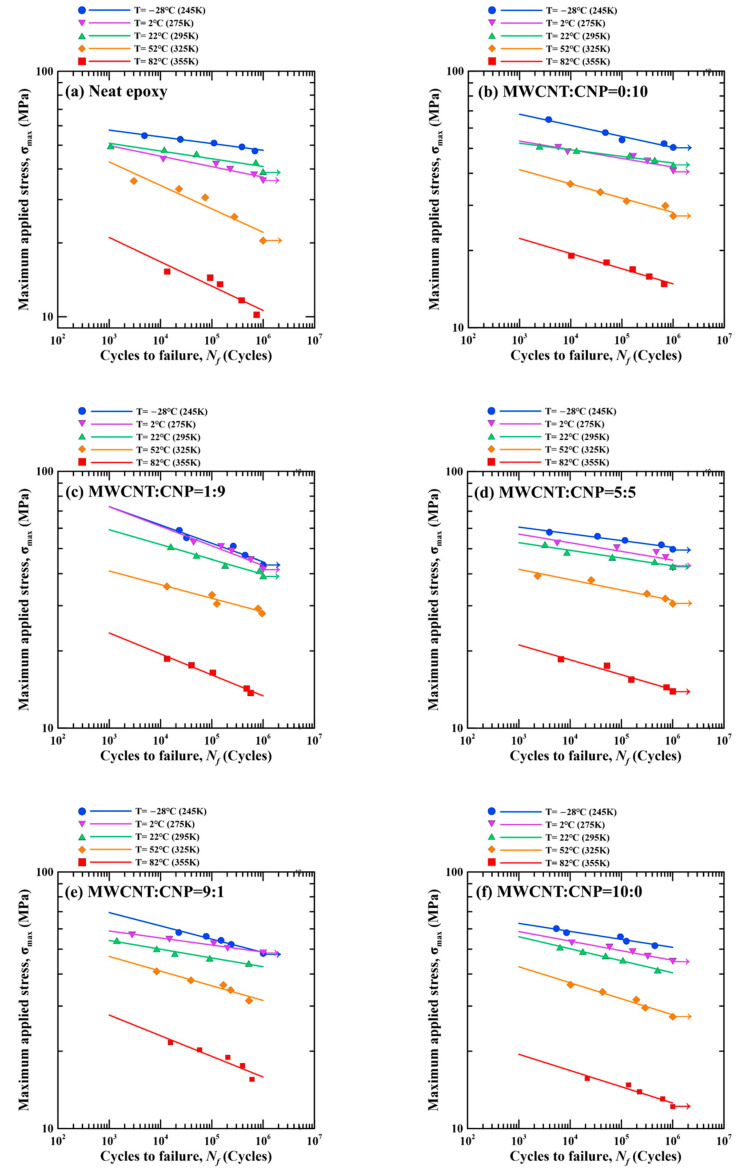
Temperature-dependent S-N curves for the nanocomposites with the MWCNT:GNP ratios of (**a**) 0:0, (**b**) 0:10, (**c**) 1:9, (**d**) 5:5, (**e**) 9:1, and (**f**) 10:0.

Table 3 lists the experimental data of the fatigue life for the nanocomposite specimens obtained in the fatigue tests conducted at different temperatures. Figure 8 shows the influence of temperature on the stress-life relationships of nanocomposite specimens with different filler ratios. A power law was used to describe the relationship between the maximum applied stress *σ*_max_ and the fatigue life *N_f_* by fitting the experimental data. The fitting model can be expressed as

*σ*_max_ = *AN_f_^B^* (2)

where *A* and *B* are the fatigue strength coefficient and fatigue strength exponent, respectively. The two parameters are dependent on the filler ratio of the specimen and test temperature. The fitting results of the parameters *A* and *B* are listed in Table 4. The fitting results are also plotted in the Figure 8, which are presented as straight lines in the log-log scale diagrams. The coefficients of determination *R*^2^ for all fitting curves are higher than 0.7, indicating that the proposed power law can model the stress-life relationship appropriately.

Figure 8 shows that, no matter what filler ratio was employed in the specimen preparation, the fatigue strength of the nanocomposite specimens decreases with the increasing temperature. The phenomenon that the fatigue strength decreases with temperature becomes more significant when the ambient temperature is higher than 22 °C. Moreover, the slopes of the S-N curves obtained at the temperatures above room temperature are deeper than those obtained at the temperatures below room temperature, indicating that the fatigue life of the studied nanocomposites is sensitive to the loading levels at low temperatures.

To further investigate the temperature-dependent fatigue properties of the studied nanocomposites, the fatigue strength for a specific fatigue life, defined as the maximum applied stress corresponding to the specific number of cycles to failure, was employed in the subsequent analysis. The four fatigue strengths, i.e., S_10^3^_, S_10^4^_, S_10^5^_, and S_10^6^_ corresponding to four specific fatigue lives of 10^3^, 10^4^, 10^5^, and 10^6^ cycle, are obtained based on Equation (2) and listed in Table 5.

**Figure 9:**
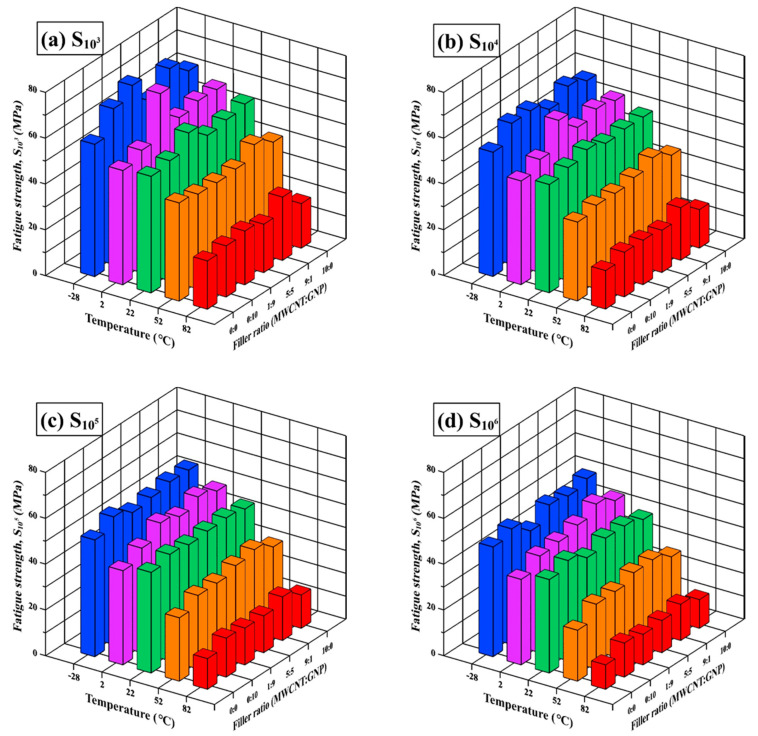
Variation of synergistic indexes for four fatigue strengths, i.e., (**a**) S_10^3^_, (**b**) S_10^4^_, (**c**) S_10^5^_, and (**d**) S_10^6^_ of the studied nanocomposites on the temperatures.

The variation of these fatigue strengths with the employed filler ratios in specimen preparation and the environmental temperatures are shown in Figure 9. Under most controlled temperature conditions, the nanocomposites with individual types of nano-filler can improve the fatigue strength of neat epoxy. Furthermore, the trinary nanocomposites with some specific filler ratios demonstrate higher fatigue strength than the ones with individual types of nano-filler. Especially the nanocomposites with a MWCNT:GNP ratio of 9:1 show remarkable improvement in fatigue strength compared with the binary ones with individual type of nano-fillers and trinary ones with other filler ratios. The increase rate in fatigue strength (based on the one of the neat epoxies) of the nanocomposites with a MWCNT:GNP ratio of 9:1 distinctly rises with increasing temperatures. In 2012, Chatterjee et al. [47] reported that the epoxy nanocomposites with a CNT:GNP ratio of 9:1 have higher flexural strength and fracture toughness than those with other filler ratios at room temperature. Other studies also found that adding rich CNTs and few graphene-based particles in the matrix can improve the mechanical properties of polymer nanocomposites [24,26,31,44,46,49]. Inserting bent-tube like MWCNTs between the sheet-like GNPs was found to prevent the agglomeration of GNPs [23,24,25], and the bridging effect caused by MWCNTs enhanced the efficiency of load transfer. The 3-D nano-filler clusters or bundles constituted by MWCNTs and GNPs form obstacles to crack growth, and subsequently improve the fatigue strength. Similar to the improvement mechanism of monotonic properties, the softening of the polymer matrix caused at high temperatures makes it easy to change the nano-fillers’ directions to the ones beneficial to load transfer.

The synergistic indexes for fatigue strengths were calculated to evaluate the synergistic effect of hybrid fillers on the temperature-dependent fatigue strengths of the studied epoxy nanocomposites. Here, the synergistic indexes for four fatigue strengths (S_10^3^_, S_10^4^_, S_10^5^_, and S_10^6^_) were calculated using Equation (1). The obtained synergistic index data for the fatigue strengths of the studied nanocomposite specimens at various temperatures are listed in Table 6.

**Figure 10:**
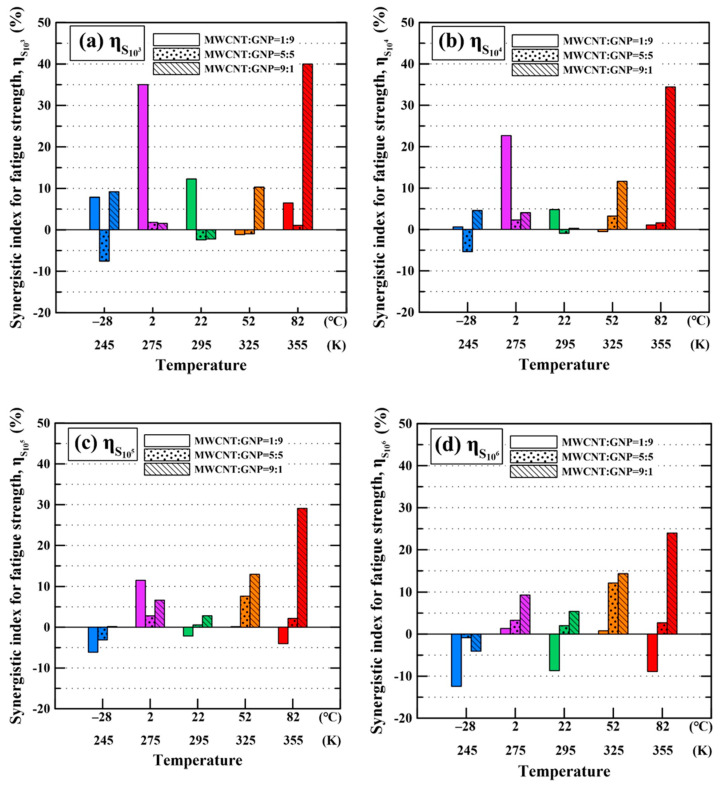
Variation of synergistic indexes for four fatigue strengths, i.e., (**a**) S_10^3^_, (**b**) S_10^4^_, (**c**) S_10^5^_, and (**d**) S_10^6^_ of the studied nanocomposites on the temperatures.

Figure 10a–d shows the variation of the synergistic indexes *η* for the fatigue strengths S_10^3^_, S_10^4^_, S_10^5^_, and S_10^6^_, respectively. In general, the specimens with the MWCNT:GNP ratios of 1:9 and 9:1 display higher synergistic effects on the fatigue strengths than those with a MWCNT:GNP ratio of 5:5. The synergistic effect on the fatigue strength for the specimen with a MWCNT: GNP of 1:9 increases with the temperature when the temperature changes from to −28 to 2 °C. When the temperature is higher than 22 °C, the synergistic effect on fatigue strength for the specimen with a MWCNT: GNP of 1:9 begins to decay. The specimen with a MWCNT: GNP of 9:1 displays higher synergistic effect on the fatigue strength with increasing temperature when the temperature is higher than room temperature. Moreover, under all test conditions, the nanocomposites with a MWCNT:GNP ratio of 9:1 have the highest synergistic index of 40% on the 10^3^-cycle fatigue strength at 82 °C. It is deduced that the high temperature and high applied load facilitate the plastic deformation of polymer matrix, further altering the relative directions of nano-fillers to those beneficial to load transfer.

**Figure 11:**
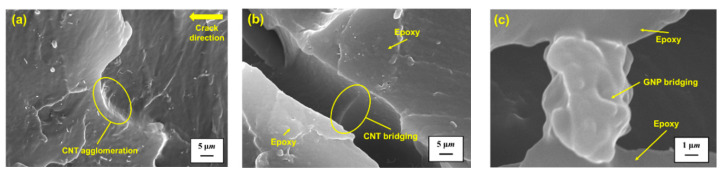
(**a**) SEM micrograph of crack deflection effect observed for the studied nanocomposite specimen with a MWCNT:GNP ratio of 9:1 tested at 2 °C (magnification 10,000×); (**b**) SEM micrograph of M WCNT bridging for the studied nanocomposites with a MWCNT:GNP ratio of 9:1 observed at 52 °C (magnification 10,000×); (**c**) SEM micrograph of GNP bridging for the studied nanocomposites with a MWCNT:GNP ratio of 1:9 observed at 52 °C (magnification 30,000×).

As mentioned above, for the epoxy nanocomposites with MWCNT and GNP hybrid fillers, the main reinforcement mechanism on the mechanical properties is the crack deflection effect. The cleavage planes are the characteristic feature of the fracture surfaces for the nanocomposites reinforced by the crack deflection effect. The crack deflection effect has been reported as the reinforcement mechanism of the mechanical properties for the polymer nanocomposites reinforced by CNTs [59], graphene-based particles [39,60,65,66,67,68,69,70,71], and hybrid nano-particles [8,72,73]. The mechanism can be applied to explain the improvement of fatigue strength of the nanocomposites. The crack front encounters the hybrid filler clusters, the crack growth is pinned, and then the plane bifurcates to bypass the obstacles, leading to the feature that the fracture surfaces ahead and behind the filler agglomeration have different heights. Compared with growing on the identical plane, crack propagation affected by crack deflection effect needs more energy dissipated to deflect the direction, and further brings up the improvement of the mechanical properties of nanocomposites. Figure 11a displays an SEM image of the fracture surface for the studied nanocomposite specimen with a MWCNT:GNP ratio of 9:1 tested at 2 °C. The crack deflection effect is verified by the presentation of the height difference of the fractures’ surface when the crack propagates through the CNT agglomeration.

**Figure 12:**
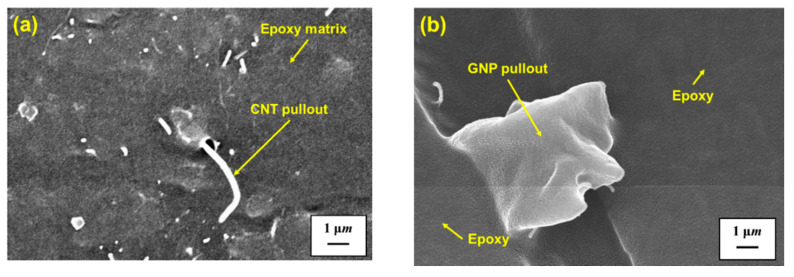
(**a**) SEM image of pullout of MWCNTs for the nanocomposite with a MWCNT:GNP ratios of 9:1 obtained in the fatigue tests performed at 82 °C (magnification 30,000×); (**b**) SEM image of pullout of GNPs for the nanocomposite with a MWCNT:GNP ratios of 1:9 obtained in the fatigue tests performed at 82 °C (magnification 30,000×).

The secondary mechanism to improve the mechanical properties of the nanocomposites is the bridging effect of the employed nano-fillers. The bridging of nano-fillers across the cracked matrix retards the further growth of crack. The bridging mechanism on the room-temperature properties has also been observed in the CNT-based nanocomposites [59,65,74,75,76], graphene-based nanocomposites [60,73,77], and hybrid nanocomposites [38,73,74,78]. Figure 11b,c show the SEM micrographs of MWCNT and GNP bridging for the studied nanocomposites with the MWCNT:GNP ratios of 9:1 and 1:9 observed at 52 °C, respectively. It is noted that whether the bridging effect is valid depends on the adhesion between the nano-fillers and the matrix. Strong wrapping the nano-fillers by the matrix implies the mechanical properties of the studied nanocomposites almost come from the strength of the nano-fillers themselves. The adhesion between the fillers and the matrix decreases significantly when the specimens are tested at high temperatures, further reducing the strength of the studied nanocomposite. Figure 12 shows the fracture surface images of the nanocomposites with the MWCNT:GNP ratios of 9:1 and 1:9 obtained in the fatigue tests performed at 82 °C, respectively. The pullout of CNTs and GNPs from epoxy polymer matrix indicates the loss of interfacial strength between the fillers and the matrix at high temperatures, implying the low strength of the studied nanocomposites at high temperatures.

**Figure 13:**
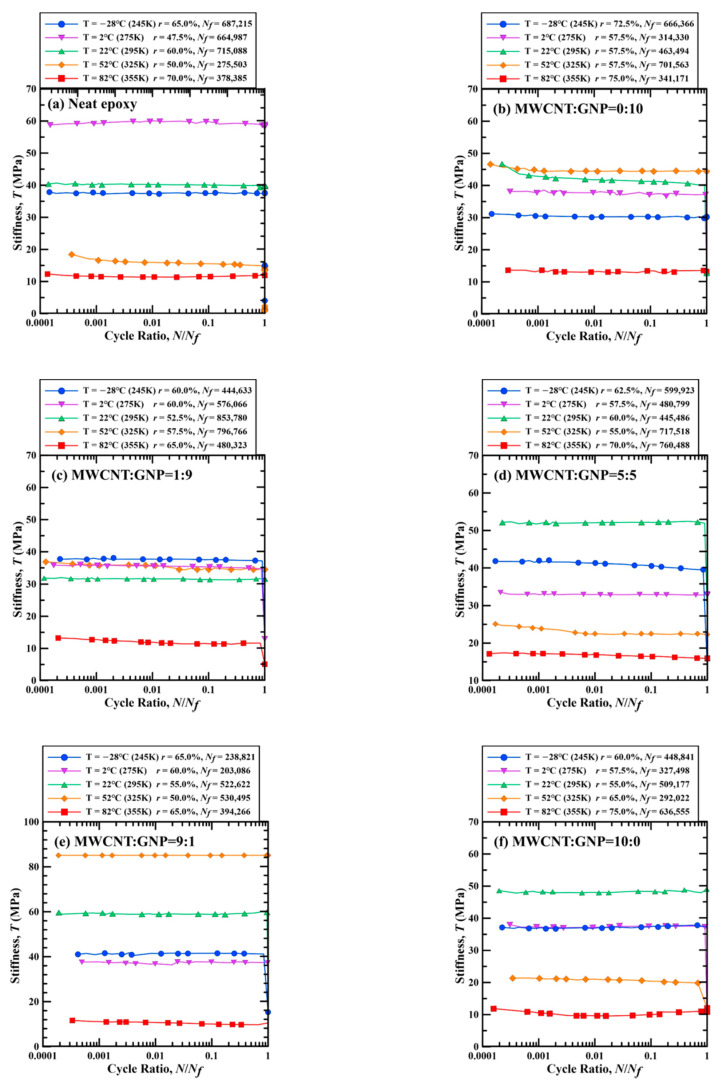
Temperature-dependent variation of specimen stiffness with the cycle ratios for the example nanocomposite specimens with the MWCNT:GNP ratios of (**a**) 0:0, (**b**) 0:10, (**c**) 1:9, (**d**) 5:5, (**e**) 9:1, and (**f**) 10:0.

Figure 13 show the examples of temperature-dependent variation of specimen stiffness *T* with the cycle ratio for the nanocomposite specimens with various filler ratios. The stiffness *T* is defined as the applied stress range divided by the strain range. The cycle ratio means the applied cycle number *N* divided by the fatigue life *N_f_*. It demonstrates that no matter what conditions of temperature and filler ratio were considered, the specimen stiffness during a fatigue test almost remains unchanged or decreases slightly except for the transient behavior in the beginning stage and the unstable behavior in the final stage. Among all considered nanocomposite specimens shown in Figure 13, the maximum drop of specimen stiffness is approximately 21% of the initial values.

**Figure 14:**
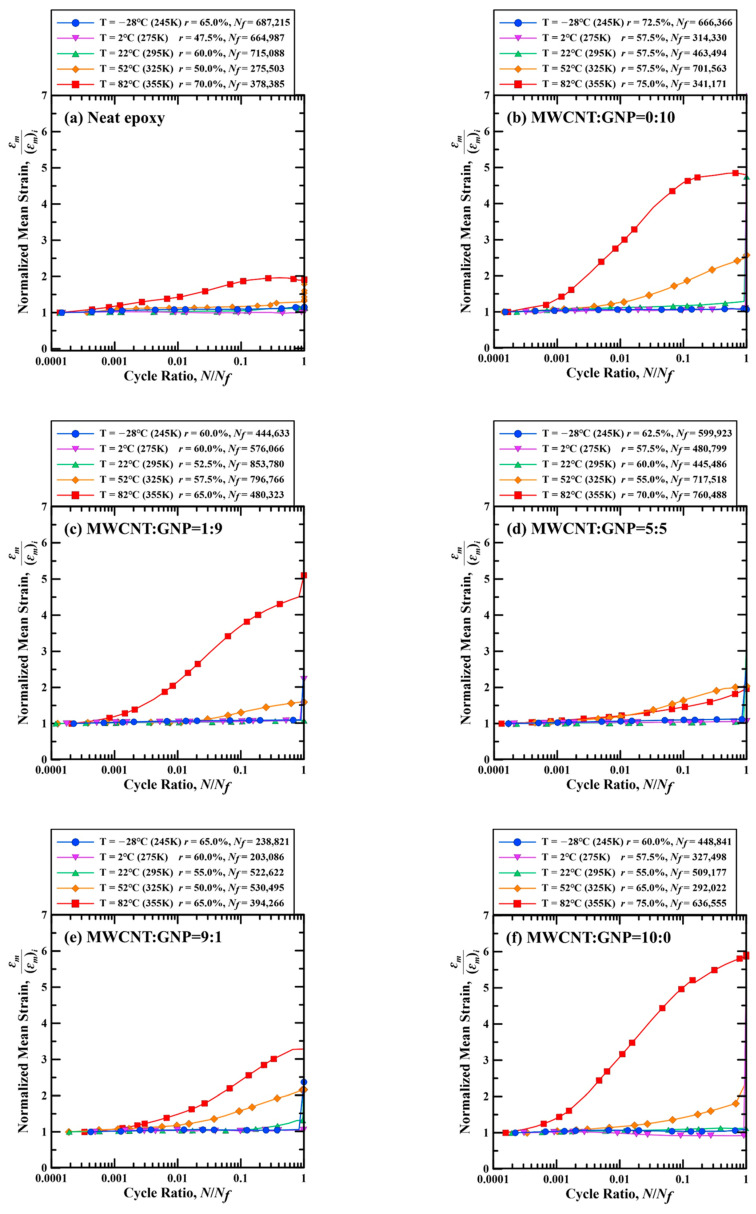
Temperature-dependent variation of normalized mean strains with the cycle ratios for the example nanocomposite specimens with the MWCNT:GNP ratios of (**a**) 0:0, (**b**) 0:10, (**c**) 1:9, (**d**) 5:5, (**e**) 9:1, and (**f**) 10:0.

Figure 14 show the examples of temperature-dependent variation of mean strain *ε_m_* with the cycle ratio for the nanocomposite specimens with various filler ratios. For the comparative purpose, the scales of the vertical axes of Figure 14a–f are identical, and the mean strain *ε_m_* is normalized by dividing by the initial value of mean strain (*ε_m_*)*_i_*. For the specimens fatigue-tested at the temperatures below 25 °C, the mean strain nearly keeps unchanged throughout the fatigue test. However, the mean strain gradually increases with the cycle ratio for the specimens tested at 52 °C, and the mean strain increases significantly with cycle ratio when the fatigue tests are performed at 82 °C. These trends of mean strain variation illustrate that the phenomenon of dynamic creep of the studied nanocomposite specimen becomes more obvious when the operating temperature increases. The variation of mean strain during a fatigue test also provides a deduction of unloading behavior of the studied nanocomposites. In general, the phenomenon of dynamic creep is observed for the specimens experiencing plastic deformation when loading. The more significant plastic behavior leads to larger residual deformation when unloading, further causes more obvious dynamic creep effect in the cyclic loading. The specimens with brittle characteristics display slight dynamic creep effect. However, the specimens with ductile feature present strong dynamic creep effect. It also explains that the nanocomposites exhibit evident dynamic creep at high temperature for their ductile property.

## 4. Conclusions

The temperature effect on the monotonic and fatigue behaviors of the epoxy nanocomposites with various MWCNT:GNP ratios have been systemically investigated in the present work. Experimental results show that environmental temperature plays an important role in the studied properties of the nanocomposites with hybrid carbon fillers. The quasi-static and fatigue strengths were found to decrease with increasing temperature substantially. Based on examination of the quantitative indexes, the nanocomposites with some specific filler ratios show a synergistic effect on the studied mechanical properties under controlled temperature conditions. The specimens with a MWCNT:GNP ratio of 9:1 display prominent performance of hybrid nano-fillers for the studied properties, and the synergistic effect rises with ambient temperature. The crack deflection effect and the bridging effect of employed fillers are the reinforcement mechanisms to enhance the studied strengths for the hybrid nanocomposites. Loss of the interfacial adhesion and pullout of nano-fillers from the matrix contribute the declination of studied strengths at high temperatures. However, the softening of matrix is beneficial to the alignment of nano-fillers in the direction of load transfer, and then improve the synergistic effect of hybrid fillers on the mechanical properties at high temperatures.

After the detailed study of temperature effect, the hygrothermal effect on the mechanical properties of the hybrid nanocomposites is suggested as the next topic of future work since the moisture is another environmental factor affecting the mechanical properties significantly. The interaction between temperature and moisture on the studied properties also needs further clarification. Due to the importance of durability and reliability of the nanocomposites in adverse environments, the aging effect is also recommended to be the candidate subject of future study.

## Figures and Tables

**Table 1:** Monotonic properties of the studied nanocomposites obtained from the tensile tests performed at different temperatures.

Filler Ratio	Monotonic Tensile Properties			
MWCNT: GNP	Tensile Modulus,	Yield Strength,	Tensile Strength,	Elongation,
	*E* (MPa)	*σ_y_* (MPa)	*σ_ult_* (MPa)	*ε_f_* (mm/mm)
**Temp. = −28 °C**				
Neat epoxy	3.38 ± 0.22	-	72.97 ± 1.51	0.029 ± 0.003
0:10	3.05 ± 0.69 (−9.8)	-	72.02 ± 2.63 (−1.3)	0.028 ± 0.002 (−3.4)
1:9	3.46 ± 0.05 (+2.4)	-	78.67 ± 0.42 (+7.8)	0.030 ± 0.001 (+3.4)
3:7	3.40 ± 0.06 (+0.6)	-	76.71 ± 1.72 (+5.1)	0.030 ± 0.001 (+3.4)
5:5	3.40 ± 0.42 (+0.6)	-	82.90 ± 1.07 (+13.6)	0.033 ± 0.005 (+13.8)
7:3	3.68 ± 0.58 (+8.9)	-	83.05 ± 2.97 (+13.8)	0.031 ± 0.003 (+6.9)
9:1	3.54 ± 0.02 (+4.7)	-	80.26 ± 1.72 (+10.0)	0.029 ± 0.001 (+0.0)
10:0	3.21 ± 0.14 (-5.0)	-	86.03 ± 1.31 (+17.9)	0.042 ± 0.008 (+44.8)
**Temp. = 2 °C**				
Neat epoxy	3.03 ± 0.10	53.83 ± 4.19	79.89 ± 1.97	0.072 ± 0.009
0:10	3.24 ± 0.09 (+6.9)	57.61 ± 3.86 (+7.0)	77.72 ± 2.78 (−2.7)	0.034 ± 0.006 (−52.8)
1:9	3.10 ± 0.20 (+2.3)	52.00 ± 2.89 (−3.4)	75.58 ± 2.14 (−5.4)	0.044 ± 0.015 (−38.9)
3:7	3.16 ± 0.11 (+4.3)	44.83 ± 2.36 (−16.7)	83.22 ± 2.27 (+4.2)	0.055 ± 0.014 (−23.6)
5:5	3.18 ± 0.17 (+5.0)	42.63 ± 0.25 (−20.8)	84.31 ± 0.92 (+5.5)	0.063 ± 0.002 (−12.5)
7:3	3.31 ± 0.23 (+9.2)	48.65 ± 3.79 (−9.6)	79.88 ± 0.12 (−0.1)	0.055 ± 0.032 (−23.6)
9:1	3.66 ± 0.10 (+20.8)	48.41 ± 3.25 (−10.1)	84.25 ± 2.93 (+5.5)	0.034 ± 0.003 (−52.8)
10:0	3.26 ± 0.16 (+7.6)	47.20 ± 4.39 (−12.3)	81.58 ± 2.36 (+2.1)	0.036 ± 0.006 (−50)
**Temp. = 22 °C**				
Neat epoxy	3.27 ± 0.13	39.54 ± 1.04	70.98 ± 0.55	0.063 ± 0.007
0:10	3.32 ± 0.22 (+1.5)	44.09 ± 4.43 (+11.5)	78.35 ± 0.39 (+10.4)	0.055 ± 0.001 (−12.7)
1:9	3.14 ± 0.34 (−4.0)	37.96 ± 10.1 (−4.0)	78.40 ± 1.30 (+10.5)	0.072 ± 0.003 (+14.3)
3:7	3.03 ± 0.17 (−7.3)	40.01 ± 3.06 (+1.2)	73.00 ± 0.31 (+2.8)	0.081 ± 0.018 (+28.6)
5:5	2.95 ± 0.33 (−9.8)	42.01 ± 4.52 (+6.2)	74.29 ± 0.39 (+4.7)	0.062 ± 0.002 (−1.6)
7:3	3.02 ± 0.11 (−7.6)	41.16 ± 3.39 (+4.1)	67.47 ± 0.52 (−5.0)	0.065 ± 0.010 (+3.2)
9:1	3.58 ± 0.35 (+9.5)	39.16 ± 4.66 (−1.0)	80.14 ± 0.77 (+12.9)	0.048 ± 0.007 (−23.8)
10:0	3.39 ± 0.31 (+3.7)	38.74 ± 7.43 (−2.0)	75.37 ± 0.73 (+6.2)	0.065 ± 0.012 (+3.2)
**Temp. = 52 °C**				
Neat epoxy	2.80± 0.46	33.14 ± 5.33	51.10 ± 1.39	0.081 ± 0.015
0:10	3.04 ± 0.28 (+8.6)	32.78 ± 2.57 (−1.1)	52.00 ± 1.05 (+1.8)	0.036 ± 0.008 (−55.6)
1:9	2.69 ± 0.06 (−3.9)	36.74 ± 4.43 (+10.9)	50.93 ± 0.51 (−0.3)	0.099 ± 0.012 (+22.2)
3:7	2.75 ± 0.26 (−1.8)	35.00 ± 3.36 (+5.6)	57.17 ± 2.24 (+11.9)	0.085 ± 0.024 (+4.9)
5:5	2.78 ± 0.10 (−0.7)	36.25 ± 0.98 (+9.4)	58.23 ± 0.56 (+14.0)	0.045± 0.003 (−44.4)
7:3	2.49 ± 0.05 (−11.1)	33.03 ± 1.17 (−0.3)	44.64 ± 0.79 (−12.6)	0.128 ± 0.043 (+58.0)
9:1	2.79 ± 0.24 (−0.4)	41.19 ± 1.65 (+24.3)	63.04 ± 0.28 (+23.4)	0.052 ± 0.016 (−35.8)
10:0	2.58 ± 0.06 (−7.9)	32.97 ± 2.25 (−0.5)	45.47 ± 1.43 (−11.0)	0.080 ± 0.060 (−1.2)
**Temp. = 82 °C**				
Neat epoxy	1.62 ± 0.09	11.83 ± 0.67	17.00 ± 1.19	0.300 ± 0.028
0:10	2.12 ± 0.36 (+30.9)	15.51 ± 1.73 (+31.1)	21.38 ± 1.54 (+25.8)	0.321 ± 0.141 (+7.0)
1:9	2.08 ± 0.33 (+28.4)	14.62 ± 2.40 (+23.6)	21.96 ± 0.58 (+29.2)	0.219 ± 0.065 (−27.0)
3:7	2.01 ± 0.34 (+24.1)	16.02 ± 0.55 (+35.4)	22.55 ± 0.86 (+32.7)	0.313 ± 0.068 (+4.3)
5:5	1.84 ± 0.06 (+13.6)	13.39 ± 0.76 (+13.2)	20.64 ± 0.49 (+21.4)	0.338 ± 0.086 (+12.7)
7:3	1.49 ± 0.17 (−8.0)	11.15 ± 1.28 (−5.7)	17.93 ± 0.65 (+5.5)	0.324 ± 0.122 (+8.0)
9:1	2.57 ± 0.24 (+58.6)	19.74 ± 0.95 (+66.9)	27.02 ± 0.84 (+58.9)	0.212 ± 0.053 (−29.3)
10:0	1.43 ± 0.40 (−11.7)	10.90 ± 2.90 (−7.9)	17.39 ± 1.39 (+2.3)	0.309 ± 0.078 (+3.0)

The number in parentheses is the percentage increase of studied property compared with the one of neat epoxy at the same temperature. MWCNT: Multi-walled CNTs.

**Table 2:** Synergistic indexes for the studied nanocomposite specimens at various temperatures.

Filler Ratio	Synergistic Index (%)			
MWCNT: GNP	Tensile Modulus	Yield Strength	Tensile Strength	Elongation
	*η_E_*	*η_σ_y__*	*η_σ_ult__*	*η_ε_f__*
**Temp. = −28 °C**				
1:9	12.851	-	7.149	2.041
3:7	9.748	-	0.639	2.041
5:5	8.626	-	4.904	12.245
7:3	16.382	-	1.495	5.442
9:1	10.833	-	−5.163	−1.361
**Temp. = 2 °C**				
1:9	−4.380	−8.077	−3.234	28.655
3:7	−2.649	−17.724	5.505	58.96
5:5	−2.154	−18.653	5.851	80
7:3	1.721	−3.325	−0.674	55.367
9:1	12.339	0.35	3.764	−5.028
**Temp. = 22 °C**				
1:9	−5.621	−12.846	0.446	28.571
3:7	−9.309	−5.826	−5.753	39.655
5:5	−12.072	1.437	−3.344	3.333
7:3	−10.359	2.02	−11.531	4.839
9:1	5.823	−0.293	5.91	−25.000
**Temp. = 52 °C**				
1:9	−10.154	12.016	−0.812	145.05
3:7	−5.238	6.587	14.246	72.764
5:5	−1.068	10.266	19.483	−22.414
7:3	−8.389	0.355	−5.880	91.617
9:1	6.245	25.004	36.678	−31.217
**Temp. = 82 °C**				
1:9	1.414	−2.851	4.666	−31.520
3:7	5.071	13.4	11.728	−1.386
5:5	3.662	1.401	6.474	7.302
7:3	−8.980	−9.224	−3.535	3.647
9:1	71.448	73.752	51.892	−31.657

**Table 3:** Experimental results of fatigue tests for the nanocomposites at various temperatures.

Temp. = −28 °C		Temp. = 2 °C		Temp. = 22 °C		Temp. = 52 °C		Temp. = 82 °C	
Max Stress	Fatigue Life	Max Stress	Fatigue Life	Max Stress	Fatigue Life	Max Stress	Fatigue Life	Max Stress	Fatigue Life
*σ*_max_ (MPa)	*N_f_* (cycles)	*σ*_max_ (MPa)	*N_f_* (cycles)	*σ*_max_ (MPa)	*N_f_* (cycles)	*σ*_max_ (MPa)	*N_f_* (cycles)	*σ*_max_ (MPa)	*N_f_* (cycles)
**Neat epoxy**									
54.73 (75)	4,879	43.94 (55)	11,353	49.69 (70)	1,074	35.77 (70)	3,024	15.29 (90)	13,436
52.90 (72.5)	24,366	41.94 (52.5)	120,927	47.91 (67.5)	11,890	33.22 (65)	22,814	14.44 (85)	93,035
51.08 (70)	109,629	39.94 (50)	227,103	46.14 (65)	50,065	30.66 (60)	73,950	13.59 (80)	144,046
49.25 (67.5)	384,766	37.95 (47.5)	664,987	42.59 (60)	715,088	25.55 (50)	275,503	11.67 (70)	378,385
47.43 (65)	687,215	35.95 (45)	>1,000,000	39.04 (55)	>1,000,000	20.44 (40)	>1,000,000	10.19 (60)	741746
**MWCNT: GNP = 0:10**									
64.82 (90)	3,691	50.52 (65)	5,710	50.93 (65)	2,449	36.40 (70)	9,837	19.07 (90)	10,222
57.62 (80)	47,485	48.57 (62.5)	8,688	48.97 (62.5)	12,930	33.80 (65)	37,871	17.96 (85)	49,923
54.02 (75)	100,396	46.63 (60)	165,947	47.01 (60)	141,794	31.20 (60)	124,484	16.90 (80)	161,109
52.22 (72.5)	666,366	44.69 (57.5)	314,330	45.05 (57.5)	436,494	29.90 (57.5)	701,563	15.85 (75)	341,171
50.42 (70)	>1,000,000	40.80 (52.5)	>1,000,000	43.09 (55)	>1,000,000	27.30 (52.5)	>1,000,000	14.79 (70)	673,865
**MWCNT: GNP = 1:9**									
59.00 (75)	23,293	52.90 (70)	44,227	50.96 (65)	15,890	35.65 (70)	13,339	18.66 (85)	13,521
55.07 (70)	32,127	51.02 (67.5)	151,277	47.04 (60)	50,147	33.10 (65)	100,218	17.57 (80)	40,325
51.14 (65)	262,364	49.13 (65)	243,229	43.12 (55)	182,751	30.56 (60)	124,922	16.47 (75)	103,670
47.20 (60)	444,633	45.35 (60)	576,066	41.16 (52.5)	853,780	29.28 (57.5)	796,776	14.27 (65)	480,323
43.27 (55)	>1,000,000	41.57 (55)	>1,000,000	39.20 (50)	>1,000,000	28.00 (55)	955,567	13.72 (62.5)	571,526
**MWCNT: GNP = 5:5**									
58.03 (70)	3,959	52.69 (62.5)	5,598	52.00 (70)	3,243	39.31 (67.5)	2,334	18.57 (90)	6,633
55.96 (67.5)	34,130	50.58 (60)	81,052	48.29 (65)	8,706	37.85 (65)	25,749	17.54 (85)	52,415
53.89 (65)	117,290	48.48 (57.5)	480,799	46,43 (62.5)	66,456	33.48 (57.5)	315,186	15.48 (75)	156,392
51.81 (62.5)	599,923	46.37 (55)	730,031	44.57 (60)	445,486	32.03 (55)	717,518	14.45 (70)	760,488
49.74 (60)	>1,000,000	42.15 (50)	>1,000,000	42.72 (57.5)	>1,000,000	30.57 (52.5)	>1,000,000	13.93 (67.5)	>1,000,000
**MWCNT: GNP = 9:1**									
58.19 (72.5)	22,687	56.87 (67.5)	2,795	54.10 (67.5)	1,406	40.98 (65)	8,405	21.62 (80)	15,572
56.18 (70)	77,595	54.76 (65)	14,879	50.09 (62.5)	8,419	37.83 (60)	39,083	20.27 (75)	58,065
54.18 (67.5)	151,919	52.66 (62.5)	108,667	48.08 (60)	18,985	36.25 (57.5)	167,016	18.92 (70)	205,828
52.17 (65)	238,821	50.55 (60)	203,086	46.08 (57.5)	90,882	34.67 (55)	233,472	17.56 (65)	394,266
48.16 (60)	>1,000,000	48.44 (57.5)	>1,000,000	44.08 (55)	522,622	31.52 (50)	530,495	15.54 (57.5)	607,591
**MWCNT: GNP = 10:0**									
60.22 (70)	5,381	53.03 (65)	10,804	50.87 (67.5)	6,339	36.38 (80)	10,240	15.65 (90)	21,696
58.07 (67.5)	8,524	50.99 (62.5)	58,374	48.99 (65)	17,633	34.10 (75)	42,976	14.78 (85)	136,663
55.92 (65)	96,073	48.95 (60)	166,549	47.11 (62.5)	49,107	31.83 (70)	195,187	13.91 (80)	227,429
53.77 (62.5)	125,387	46.91 (57.5)	327,498	45.22 (60)	107,018	29.56 (65)	292,022	13.04 (75)	636,555
51.62 (60)	448,841	44.87 (55)	>1,000,000	41.45 (55)	509,177	27.28 (60)	>1,000,000	12.17 (70)	>1,000,000

The number in parentheses indicates the loading level *r* (%).

**Table 4:** Fitting results of S-N curves at various temperatures for the studied nanocomposites.

Filer Ratio (MWCNT:GNP)	Temperature (°C)	Fatigue Strength Coefficient, *A*	Fatigue Strength Exponent, *B*	Coefficient of Determination *R*^2^
0:0 (Neat epoxy)	−28	69.584	−0.027	0.968
	2	66.636	−0.042	0.894
	22	63.457	−0.032	0.88
	52	82.523	−0.095	0.9
	82	41.755	−0.099	0.845
0:10	−28	91.476	−0.043	0.966
	2	67.491	−0.034	0.874
	22	62.672	−0.026	0.958
	52	60.419	−0.055	0.944
	82	33.525	−0.059	0.956
1:9	−28	119.689	−0.072	0.935
	2	122.992	−0.076	0.905
	22	88.34	−0.058	0.972
	52	59.098	−0.053	0.921
	82	41.304	−0.082	0.98
5:5	−28	73.043	−0.027	0.956
	2	72.06	−0.034	0.716
	22	64.911	−0.030	0.947
	52	55.214	−0.041	0.937
	82	31.71	−0.059	0.953
9:1	−28	98.834	−0.051	0.971
	2	71.017	−0.027	0.974
	22	68.564	−0.034	0.977
	52	69.561	−0.057	0.922
	82	48.329	−0.081	0.883
10:0	−28	78.239	−0.031	0.937
	2	76.129	−0.038	0.97
	22	77.195	−0.047	0.989
	52	65.945	−0.063	0.961
	82	30.253	−0.064	0.924

**Table 5:** Fatigue strengths for the fatigue lives of 10^3^, 10^4^, 10^5^, and 10^6^ cycles at different temperatures.

Filler Ratio	Fatigue Strength (MPa)			
MWCNT: GNP	S_10^3^_	S_10^4^_	S_10^5^_	S_10^6^_
**Temp. = −28 °C**				
Neat epoxy	57.74	54.26	50.99	47.92
0:10	67.97 (+17.7)	61.56 (+13.4)	55.76 (+9.3)	50.50 (+5.4)
1:9	72.79 (+26.1)	61.67 (+13.6)	52.25 (+2.5)	44.26 (−7.6)
5:5	60.61 (+5.0)	56.96 (+5.0)	53.53 (+5.0)	50.30 (+5.0)
9:1	69.49 (+20.3)	61.79 (+13.9)	54.94 (+7.7)	48.85 (+2.0)
10:0	63.16 (+9.4)	58.81 (+8.4)	54.75 (+7.4)	50.98 (+6.4)
**Temp. = 2 °C**				
Neat epoxy	49.86	45.26	41.09	37.3
0:10	53.36 (+7.0)	49.35 (+9.0)	45.63 (+11.1)	42.19 (+13.1)
1:9	72.76 (+45.9)	61.08 (+34.9)	51.27 (+24.8)	43.04 (+15.4)
5:5	56.98 (+14.3)	52.69 (+16.4)	48.72 (+18.6)	45.05 (+20.8)
9:1	58.93 (+18.2)	55.38 (+22.4)	52.04 (+26.7)	48.91 (+31.1)
10:0	58.55 (+17.4)	53.65 (+18.5)	49.15 (+19.6)	45.03 (+20.7)
**Temp. = 22 °C**				
Neat epoxy	50.87	47.26	43.9	40.78
0:10	52.37 (+2.9)	49.33 (+4.4)	46.46 (+5.8)	43.76 (+7.3)
1:9	59.18 (+16.3)	51.78 (+9.6)	45.31 (+3.2)	39.64 (−2.8)
5:5	52.76 (+3.7)	49.24 (+4.2)	45.95 (+4.7)	42.89 (+5.2)
9:1	54.21 (+6.6)	50.13 (+6.1)	46.35 (+5.6)	42.86 (+5.1)
10:0	55.79 (+9.7)	50.07 (+6.0)	44.94 (+2.4)	40.33 (−1.1)
**Temp. = 52 °C**				
Neat epoxy	42.81	34.4	27.64	22.21
0:10	41.32 (−3.5)	36.41 (+5.8)	32.08 (+16.0)	28.26 (+27.2)
1:9	40.98 (−4.3)	36.27 (+5.4)	32.11 (+16.1)	28.42 (+27.9)
5:5	41.60 (−2.8)	37.85 (+10.0)	34.44 (+24.6)	31.34 (+41.1)
9:1	46.92 (+9.6)	41.15 (+19.6)	36.09 (+30.6)	31.65 (+42.5)
10:0	42.68 (−0.3)	36.91 (+7.3)	31.93 (+15.5)	27.62 (+24.3)
**Temp. = 82 °C**				
Neat epoxy	21.07	16.78	13.36	10.63
0:10	22.30 (+5.8)	19.47 (+16.1)	17.00 (+27.3)	14.84 (+39.5)
1:9	23.44 (+11.2)	19.41 (+15.7)	16.07 (+20.3)	13.30 (+25.1)
5:5	21.10 (+0.1)	18.42 (+9.8)	16.08 (+20.4)	14.03 (+32.0)
9:1	27.62 (+31.1)	22.92 (+36.6)	19.02 (+42.4)	15.78 (+48.4)
10:0	19.44 (−7.7)	16.78 (+0.0)	14.48 (+8.4)	12.50 (+17.5)

The number in parentheses is the percentage increase of studied property compared with the one of neat epoxy at the same temperature.

**Table 6:** Synergistic indexes for the fatigue strengths of the studied nanocomposites at different temperatures.

Filler Ratio	Synergistic Index for Fatigue Strength (%)			
MWCNT: GNP	$\eta_{S_{10^{3}}}$	$\eta_{S_{10^{4}}}$	$\eta_{S_{10^{5}}}$	$\eta_{S_{10^{6}}}$
**Temp. = −28 °C**				
1:9	7.853	0.622	−6.130	−12.434
5:5	−7.547	−5.355	−3.129	−0.869
9:1	9.191	4.581	−0.159	−4.084
**Temp. = 2 °C**				
1:9	35.029	22.705	11.504	1.325
5:5	1.819	2.31	2.801	3.292
9:1	1.55	4.066	6.644	9.285
**Temp. = 22 °C**				
1:9	12.267	4.816	−2.161	-8.693
5:5	−2.441	−0.923	0.56	2.006
9:1	−2.235	0.266	2.81	5.396
**Temp. = 52 °C**				
1:9	−1.150	−0.507	0.138	0.783
5:5	−0.959	3.243	7.614	12.161
9:1	10.297	11.63	12.976	14.334
**Temp. = 82 °C**				
1:9	6.47	1.081	−4.037	−8.897
5:5	1.066	1.608	2.149	2.69
9:1	39.99	34.44	29.109	23.987

## Data Availability

Data sharing not applicable.
